# Superiority of High Potencies over Low in the Treatment of Worst Forms of Disease

**Published:** 1881-07

**Authors:** Rollin R. Gregg

**Affiliations:** Buffalo, New York


### SUPERIORITY OF THE HIGH OVER THE LOW POTENCIES, IN THE TREATMENT OF THE WORST FORMS OF DISEASE.

ROLLIN R. GREGG, M. D., BUFFALO, NEW YORK.

IF a great superiority of the high over the low potencies, in the treatment of the worst forms of disease can be shown, then, certainly, they are shown to be best in the treatment of all forms of disease, as the greater contains the less in all departments of Nature. And to show the greater efficacy of the high potencies in the worst diseases, I will take acute hydrocephalus, and acute encephalitis, without dropsical effusions. These, certainly, are among the worst possible forms of disease, not alone in their fatality, but in the terrible sufferings attending them. Indeed, no other acute diseases can be named that are so uniformly fatal as these, or in which the sufferings are greater. Consequently, if a course of treatment can be pointed out that is quite commonly successful in those diseases, that treatment should be followed, at least till something better is found.

To cover the grounds taken above, as fully and carefully as I wish, I must take a retrospective view as follows: I commenced the practice of medicine in the spring of 1853. From that time until February, 1865, or for about twelve years, I never cured, or saw cured, a case of fully established acute hydrocephalus, or of fully established inflammation of the brain without effusion, although I treated numerous cases of both, and was called in consultation by other physicians in several cases. During all of that time, however, I prescribed the low potencies from the third to the sixth, generally two remedies in alternation, and doses at one to two hours intervals, not daring to trust such cases to any higher potencies, or to longer intervals between doses.

In the fall of 1864, I attended an important case of acute hydrocephalus, administering low potencies, mostly the third, and in this case, at intervals of half-an-hour, part of the time, to see if I could not, by that means, make some favorable impression upon the disease. My patient died, however, as had all similar cases I had ever treated. But as it endured the most extreme sufferings for several days before life was taken, I made up my mind that such, or, at least, that some of such cases ought to be, and could be, cured, providing the proper treatment were resorted to early in the disease. And I then resolved, if ever called to another such case, to treat it upon the strictest principles laid down by Hahnemann, or with high potencies, and the doses at long intervals.

As strange as it may appear, the very next case of the kind I was called to, which was on the 22d of the next February—that is, February, 1865—the patient, a boy of three or four years, had been first under treatment for acute hydrocephalus, for about a week, by one of our most prominent old school physicians, who gave him up to die, saying such cases were utterly incurable; and then one of our homœopathic physicians was called, and attended him two or three days, and said he could do nothing for the patient; then I was called.

I found the child lying utterly unconscious and sightless, with both eyes turned in, so much that the angle of vision of each eye, could it have seen, was at right angles with the other across the nose, the eyes wide open, and no flinching from placing the finger directly down upon the ball over the pupil. All other indications of the case were equally serious; and, of course, I gave no encouragement whatever of cure. The parents, however, wished me to prescribe, and I did so.

Here, then, was an opportunity to test a different method of treatment, where little or no responsibility could attach, providing nothing were done to hasten the patient's death. Consequently, after getting all the facts I could, as to how the child was taken and had been, I prescribed *Calc. Carb.* 6000th, one dose, and awaited developments for twenty-four hours, when there was some, though not very marked improvement, and the case was allowed to go on another day upon the one dose of *Calcarea.* At the end of that time, or forty-eight hours after giving the dose of *Calc.*, there was quite a perceptible change of symptoms, for which one dose of *Nux Vomica* 2000th was administered and results awaited. The next day found my patient clearly better in every respect; so there could be no doubt about allowing him to go another day without medicine, when still greater improvement was manifested; no more medicine was given; consciousness returned in a day or two after, and on the eighth day of my attendance, I dismissed the boy cured, and he continued well for several years after, or until I lost sight of the family.

The boy's right eye recovered its natural position, but the left remained permanently turned towards the nose, the last I knew.

The succeeding fall I was called into the same neighborhood to a boy of six years of age, who had been suffering ten days from inflammation of the brain, evidently without effusion, and had been under the care, from the first, of a homœopathic physician, who had been giving low potencies and doses at short intervals. This patient, too, was entirely unconscious and had been several days; and in addition had had severe convulsions, which increased in frequency and severity until a day or two after my being called, when he had successive convulsions for one entire day, and so severe that once, when his attendants left him for a minute or two, he was so suddenly and so violently convulsed, that he was thrown clear of the bed upon the floor. Constipation had existed from the first, and he had one peculiar symptom that may be of interest to mention. This was a continual boring with the left forefinger under the right *ala nasi* until he bored a hole a third to half an inch in diameter entirely through the lip at that point, on the teeth and gum. All efforts to hold or bind his hand would at once bring on a convulsion, so that we had to desist from that, and allow him to go on with this work.

The treatment was entirely with the high potencies—the 2000th and upwards, and doses at not less than twelve hours interval on two or three occasions, while all the rest of the time they were given at twenty-four to forty-eight, or more, hours intervals. The remedies administered were *Nux Vomica*, *Hyoscyamus*, *Cuprum*, *Helleborus*, *Belladonna*, etc., but none of these appeared to have the least effect until *Belladonna* was prescribed, after the first dose of which, in the 2000th potency, the patient improved steadily and markedly for two or three days, when a second dose was given, which entirely completed the cure of the case, and I discharged the patient well, excepting some remaining debility, in ten days to a fortnight from my first call. Nor was there any impairment of mind, defects of vision, or other annoying sequelæ of the case, but a complete restoration to health, which is a no less remarkable and happy result of such treatment, than the safe relief at the time of such violent symptoms.

The next case I will relate, occurred in May, 1867, in a boy aged four years, whom I had charge of from the commencement of his sickness. For the first week or ten days, his symptoms were obscure but then rapidly developed into one of the most violent cases of hydrocephalas that I have ever seen. During that week or ten days, I prescribed several remedies, at intervals of twelve to twenty-four hours, and in the single or at most second dose, but without relief. By the end of that time, the symptoms became simply frightful. Those peculiar intermitting screams of hydrocephalus, with which all are familiar who have seen the worst forms of the disease, were more or less continued night and day at intervals of a few minutes, and could be heard for some distance on the street. But what was even much worse than that, were the constant struggles of the patient for hours together, rising upon his hands and knees in bed, and then plunging head first as far as he could, or was allowed to, without the slightest reference to where or what his head might strike. Of course, by this time he had become thoroughly delirious and unconscious of what he did; but to restrain him from his efforts, aside from guarding his head from injury, greatly aggravated all his other symptoms; consequently, a row of large pillows was placed entirely around his bed and secured there to protect him as well as we could in that way, and he was then allowed to take his own course. He would rise and plunge his head into the pillows, then rise at once and plunge again; and so on, continuously, for two or three hours at a time, until utterly exhausted; then he would rest a little while, a half hour or so, when he would renew these struggles and go plunging around and around the bed again for hours. In this way he wore the skin entirely through on all the projecting parts of his face, as upon the point and ridge of his nose, the cheek-bones, the brow and upper part of his forehead, ears, chin, sides of the under jaw, etc.

At an early call one morning, after these struggles had been going on thirty-six to forty-eight hours and *Belladonna*, *Hyoseamus*, *Nux Vomica*, etc., in the single dose, having failed to relieve, I inquired, as I had done a day or two previously, as to the excretions from his kidneys, and found there had been no emission of urine from the previous afternoon, and that very scanty, and very dark colored; or, at least it stained the bedding deeply. This determined at once my choice of the remedy, which was *Helloborus*, one dose of which in the 1000th protency, was then given. Visited my patient again at 2 P. M., and found there had been a free passage of urine, but as yet there was no mitigation of the violence of the brain symptoms, the rising and plunging headlong continuing as violent as before. There was no doubt in my mind, however, that the medicine was acting favorably, in its effects upon the kidneys, and that a few hours more would show relief of brain symptoms; so I did not administer more medicine to disturb the action of the one dose. That evening justified my highest hopes; for, not only had the patient passed urine freely two or three times, but had some perspiration, (the first for several days), and had slept quietly two or three hours.

Under these circumstances, no more medicine was given, of course, and he was left for the night without more. The next morning I found his brain symptoms had been very severe part of the night, but better than the night previous, and as the kidneys still continued free in their action, I concluded to still give no more medicine. The next day showed great improvement in most respects, though he was yet delirious and unconscious, and had plunged about considerably at times during the night; but he was still allowed to go on the one dose of *Helleborus*. Twenty-four hours later all delirium and struggles had ceased, and his kidneys continued active; but he was yet unconscious, and from some change in the symptoms, I was led to give him one dose of *Pulsatilla*, 1000th protency, which was allowed to act three days, with gradual improvement from day to day, though I was in great doubt whether it acted or accomplished more than the *Hellebore* have done by its continued action. In the meantime, he had come to partial consciousness; but from an ulcerating eruption and swellings something like boils that now began to develop upon the scalp, I gave one dose of *Sulphur* in the 6000th potency; and either under this or the still continued effects of *Hellebore*, all the brain symptoms named ceased in another week, and convalescence was established. But this was by no means the end of the case.

From a talkative, active boy, his disease made him completely dumb for the time being. He never uttered a word for six weeks from the time he was able to leave his bed. He seemed to lose all knowledge of the use of language, but began to regain it gradually after the six weeks, by playing with other children. Words came to him singly, as to a baby; then in groups of two or three, until finally, after many weeks, he fully recovered his faculties in this respect, and then talked almost constantly when awake for several weeks.

During all this time, I felt that a permanent injury had been done the brain; that insanity or idiocy would certainly follow, and that it would have been much better had he died, instead of being saved for such a fate. But I continued the treatment, by giving a single or second dose once in three or four days to a week or more, of various remedies in succession that seemed best indicated, until an entire recovery from every morbid condition of body and mind followed. And from that time, he has always been the brightest scholar in all his classes at school up to last year, when he graduated with the highest academic honors ever given at the institution.

Thus it is always when we follow Nature's laws in the treatment of disease, and, at the same time, avoid over-treatment, the good results follow for years and often for life-time; whereas, neglect of the law and over-treatment, always result either in the greatest disaster to the patient at the time, or in sad consequences to health for years, and often for a life-time. For instance, supposing that I had prescribed morphine or other anodynes to have kept that boy from his struggles that were so terrible to witness, death would have been inevitable within two or three days; or, had I administered doses of homœopathic remedies every two or three hours as I had done formerly, he would have died, as all previous similar, but less severe, cases had that I ever treated in that way.

The next case I was called to, was three months subsequent to the last, or in August, 1867. This was a child eight or ten months old, who had been given up to die of hydrocephalus, by a council of some of the best old-school physicians of our city. The attending and consulting physicians all told the father that there was absolutely no cure for such cases after effusion had once taken place. It was forty-eight hours after they proclaimed effusion fully established, that I was called. The child then lay entirely unconscious and had, for the two days; pupils enormously dilated, with no flinching upon placing the finger upon the eye-ball, and there was every other indication of rapidly approaching death.

The first question I asked was, "Has there been any action of the kidneys for a day or two?" and upon being assured there had been little or none, I at once prescribed *Helleborus*, 1000th; one dose. This was followed, in a few hours, with a quite free discharge of urine, which was increased and maintained subsequently, with a modification of all brain symptoms day by day, until consciousness returned in about a week, and an entire restoration to health in two or three weeks. In all this time, however, only one more dose of *Helleborus* was given, or two doses in all of it, besides a single dose each of *Bell.*, *Nux.*, and one or two other remedies in the high potencies, for symptoms calling for them; but in no case was any of these doses administered short of twenty-four hours, and generally at intervals of two to four or more days. This child, too, developed as the brightest one of a large family of children, and an ornament in the schools she has attended.

Another case was that of a little girl of eighteen months, who was rendered unconscious from hydrocephalus, and a few days subsequently complete hemiplegia, left side, resulted, despite several remedies. The next day, after paralysis was established, I prescribed *Belladonna*, in the 2000th potency, one dose of which cured the hemiplegia completely in two or three days, and a single dose of two or three other remedies, administered for other symptoms that arose later, entirely restored the child to health in two or three weeks.

The next case of interest was one where I was called in consultation by another physician, the symptoms and conditions resembling quite closely, when I was called, the third case reported in this series, but had not been so long developing into a violent case, nor was the convalescence as protracted. There was, however, the same terrible screaming, rising upon the hands and knees, and plunging headlong upon the bed, hour after hour. Finding there had been excretion of urine for two or three days, I advised *Helleborus*, in the 1000th potency, one dose of which was given with marked effect by the next day, and another dose of that a day or two later, and a dose or two of a few other remedies, entirely restored that boy to full health in a few weeks.

Still another case, as bad as any here reported, excepting the third, that is, less demonstrative than that, but where more profound stupor resulted after a few days screaming, was a boy, three or four years old, who was cured by a dose or two each of *Nux Vomica*, *Helleborus*, and one or two other remedies in the 1000th or 2000th potencies, and doses at not less than twenty-four to forty-eight or more hours apart.

The last case I will now cite, was that of a boy of ten or twelve years, who, until he became unconscious, complained of all or nearly all his pain being in the upper part of his occiput. In this case *Lycopodium* 6000th, two doses, was the curative remedy, after several others had failed, and the patient is now living in the best of health at the age of twenty, or a little upwards.

Now, under any treatment that I have ever seen or known, besides that detailed in these cases, every one of these patients must almost certainly have died. And I ask, in all candor, is it not about time that physicians of our school should more generally test this method of treatment for themselves, and thereby learn its efficacy, rather than continue that which we all know offers but little or no hope.

In conclusion, let me say to young physicians, be extremely cautious about giving encouragement in brain diseases of children, where they have received a fall upon the head that has probably resulted in a rupture of a blood-vessel and a coagulum of blood in the substance of the brain. These are necessarily fatal, and I have seen five or six children die in that way, the last one but a few weeks ago. In not one of these cases did I ever see any, or but little relief given by medicine. Also be cautious in giving encouragement in brain diseases that succeed immediately upon very exhausting or protracted diarrhœas, protracted and exhausting attacks of whooping cough, etc. In these cases the vitality is often so completely exhausted before, or by the time the brain disease develops, that there are no latent forces left in the system that can be called out or aroused by medicine, to thereby restore the patient to health. Although in several of the cases here given, the brain disease was preceded by very exhausting diarrhœa, and notably so the case taken from the hands of the council of old-school physicians.

